# Prevention of Hair Heat Damage via Thermoresponsive Organic Silicon-Modified Keratin

**DOI:** 10.3390/molecules31030521

**Published:** 2026-02-02

**Authors:** Chaohai Li, Jinhua Li, Kuan Chang, Jing Wang

**Affiliations:** Key Laboratory of Synthetic and Biological Colloids, Ministry of Education, School of Chemical & Material Engineering, Jiangnan University, Wuxi 214122, China; ocean_l08@163.com (C.L.); jinhua.li@jnmwht.com (J.L.)

**Keywords:** thermoresponsive, hydrolyzed wool keratin, 3-[(2,3)-Epoxypropyl] propyl methyldimethoxysilane, heat damage protection

## Abstract

Heat damage is a common phenomenon that often occurs when drying and straightening hair. After heat damage, the hydrophobic barrier on the hair’s surface becomes disrupted, thereby altering the hair’s hydrophilicity. Meanwhile, during the process of heat damage, the rupture of the hair’s cuticles causes the hair to become dry and rough, with a decrease in gloss and a decline in mechanical properties. This study utilized epoxy silane and hydrolyzed wool keratin to synthesize a thermally responsive organic silicon-modified keratin (OSK) to prevent hair from heat damage. OSK was synthesized from epoxy silane and hydrolyzed keratin, with yield determined by quantifying free amino groups. Its hair-care performance was evaluated through assessments of hair surface morphology, mechanical properties, and optical gloss, and by combing test and contact angle measurements. Mechanisms underlying surface hydrophobicity and hair scale protection were investigated using FTIR, XPS, and DSC. Specific performance parameters were evaluated using a single-fiber strength tester and a multifunctional hair-testing instrument. FTIR confirmed successful covalent grafting, with synthesis optimized to a 90.67% yield. OSK forms a protective film on hair surfaces, verified by SEM, XPS, and TEM, restoring damaged hair hydrophobicity to a 117° contact angle and enhancing thermal protection to 136° upon heating. Beyond hydrophobic-barrier restoration, OSK improved hair gloss by 30.26% and reduced frizz by 39.33%, while restoring the key performance of virgin hair. It also provides exceptional water-repellency protection and sensory enhancement. Under thermal stress, the protective film mechanically increased tensile strength by 6.58% and yield zone tensile force by 4.65%. This article demonstrates that OSK is an effective heat-sensitive agent. When damaged by heat sources such as hair dryers, it will form a protective film on the surface of the hair, thereby protecting the surface properties of the hair.

## 1. Introduction

The hair shaft consists of three distinct regions, which are, from the periphery inward, the cuticle, the cortex, and the medulla [1]. As the outermost layer, the cuticle consists of cells measuring 0.3–0.5 μm in thickness with a visible length of 5–10 μm. This imbricate arrangement is widely regarded as the primary determinant of the frictional properties of the hair. At the molecular level, the hair cuticle is a keratin–lipid structure, which provides a certain barrier effect to prevent hair damage [2].

However, frequent use of hair dryers and various thermal styling tools progressively compromises the structural integrity of hair fibers, leading to cumulative damage over time [3,4]. This heat-induced damage can be primarily categorized into two distinct types based on the context and mechanism of application.

The first type of damage originates from the post-wash drying process. During blow-drying, sustained exposure to hot airflow causes a rapid temperature increase on the hair surface [5]. Research by Petrovicova et al. [6] demonstrated that even blow-drying at 70 °C induces a sharp temperature rise resulting in sustained cuticle damage. The underlying mechanism involves the penetration of hot air beneath the cuticular scales, causing them to lift and expand, while subsequent moisture evaporation further promotes progressive embrittlement of the hair [6,7,8]. The second type of damage arises from the use of styling tools like a flat iron. This form of contact-based, high-temperature heating delivers intense thermal shock to localized sections of hair within a very short duration. During this process, the cuticles undergo repeated expansion and contraction from heating and cooling cycles, leading to edge lifting and even delamination [9]. Such physical disruption directly damages the critical hydrophobic lipid layer anchored to the cuticle surface, notably 18-methyleicosanoic acid (18-MEA) [6]. The loss of 18-MEA fundamentally alters the surface properties of hair, shifting it from a hydrophobic to a hydrophilic state, thereby compromising its natural barrier function. The structural damage resulting from these two types of thermal assault—including physical cuticle impairment, loss of the hydrophobic barrier, and accelerated depletion of internal keratin and lipids [10]—collectively contributes to a series of adverse alterations in hair: increased surface friction, diminished mechanical strength, and resilience, ultimately manifesting as dryness, brittleness, susceptibility to breakage, and loss of gloss [11].

To repair surface damage on the hair or provide protection, a variety of functional components have been extensively studied. Traditional conditioning agents, such as polymers, oils, waxes, hydrolyzed amino acids, and cationic molecules, mainly achieve hydrophobicity and improve combing performance by lubricating the hair surface and neutralizing negative charges [12,13,14,15]. Among them, silicone oil, as the core component of hair conditioners, has been fully confirmed for its functions of repairing the hair surface, maintaining hydrophobicity, and providing lubrication. In the field of active protection against heat damage, research focuses on developing components that can enhance the heat stability of hair. Stiefel et al. [16] systematically elaborated on the applications and mechanism of action of antioxidants, silicone oils, and other chemical substances for improving the heat-protection efficacy of hair. However, these hydrophobic or non-ionic functional components (such as traditional silicone oil) encounter a fundamental challenge in practical applications: they have difficulty effectively depositing on the negatively charged hair surface and forming a stable protective film [12,15]. Nazir et al. [15] clearly indicates that due to the mismatch between the hydrophobic nature of silicone oil and the electrical properties of the hair surface, its direct adsorption effect is poor.

The hydrolysis of keratin is widely studied due to its high similarity in amino-acid composition and structure to human-hair keratin. This similarity is utilized to enhance its bioavailability and repair performance in hair care. Such homology endows it with unique biological affinity and low allergenicity [8,17]. The behaviors and mechanisms of hydrolyzed keratins with different molecular weights in hair show significant differences. The research by Malinauskyte et al. [17] indicates that low molecular weight (approximately 221 Da) hydrolyzed keratins can penetrate deeply into the hair’s cortex layer by virtue of their smaller hydrodynamic radius, thereby increasing the cross-sectional area and volume of the hair strands; while medium-to-high molecular weight (such as approximately 75,440 Da) keratin peptides mainly adsorb on the hair surface to form a protective film, effectively enhancing Young’s modulus of hair and reducing fiber breakage. This molecular weight-dependent penetration and deposition pattern provides a basis for the targeted design of repair products. Fan et al. [18] demonstrated that the hydrolysis of keratin can form a film on the surface of the hair, acting as an UV attenuator to reduce the surface-morphology damage caused by photoaging. Under UV radiation, the keratin in the film will be degraded into smaller peptides and amino acids. These fragments can penetrate deeper into the hair shaft, enhancing the chemical-bond strength within the fibers, thereby improving the stretching performance of the hair after UV radiation. This is consistent with the conclusion of Barba et al. [9] that keratin peptides can repair the moisture content, stretching performance, and water absorption of damaged hair.

In the traditional silicone-oil protection system, hair is mainly protected through physical adsorption. The silicone oil adheres to the surface of the hair to form a protective film, thereby protecting the hair’s hydrophobic barrier and gloss. However, there are problems with difficulty in dissolution and weak adsorption force in its application. Based on this, this study proposes to develop a material by modifying the traditional silicone-oil adsorption system. Compared with the traditional silicone oil, it has better water solubility, combines with the keratin that is both water-soluble and hair-friendly, uses heat-triggered Si-OH dehydration cross-linking, and can actively respond to heat stimulation. It forms a network membrane structure to enhance the barrier effect. At the same time, it also has the biological affinity and repair function of keratin. It overcomes the deposition problems of the two materials. This is expected to achieve more efficient heat protection.

As reported by Haidan Zhao et al. [19], a thermally stable resin coating was developed using (3-aminopropyl)triethoxysilane as a cross-linker. The incorporation of Si–O–Si bonds enhanced thermal resistance, attributed to the high bond strength of siloxane linkages [20,21]. A modified keratin was successfully synthesized using hydrolyzed wool keratin and 3-[(2,3)-epoxypropyl]propyl methyl dimethoxy silane (GPMDMS), with structural confirmation provided by FTIR spectroscopy and free amino group quantification. Comprehensive evaluation through scanning electron microscopy (SEM), differential scanning calorimetry (DSC), X-ray photoelectron spectroscopy (XPS), Transmission electron microscopy (TEM), combing tests, friction measurements, and tensile analysis confirmed its efficacy and elucidated the functional mechanism.

## 2. Results and Discussion

### 2.1. The Synthesis Route of OSK

**Figure 1:**
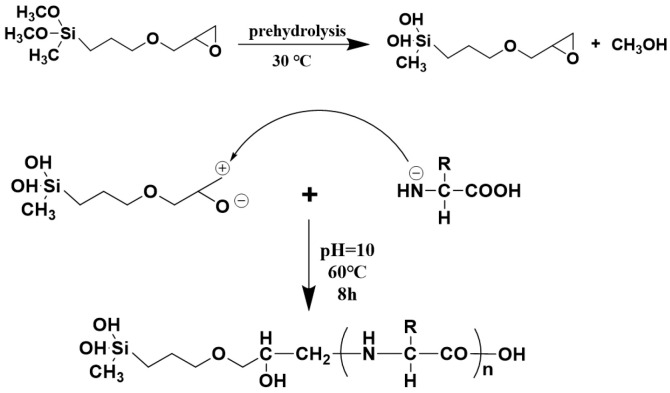
Synthetic route of OSK.

The synthesis route of organic silicon-modified keratin (OSK) is illustrated in Figure 1. After hydrolysis, epoxy silane yields hydroxyl groups. Under alkaline conditions (with a pH value of 10), the epoxy groups of the silane coupling agent react with the nucleophilic amino groups present on the keratin chain to form a ring-opening reaction, resulting in the final functionalized keratin product.

**Figure 2:**
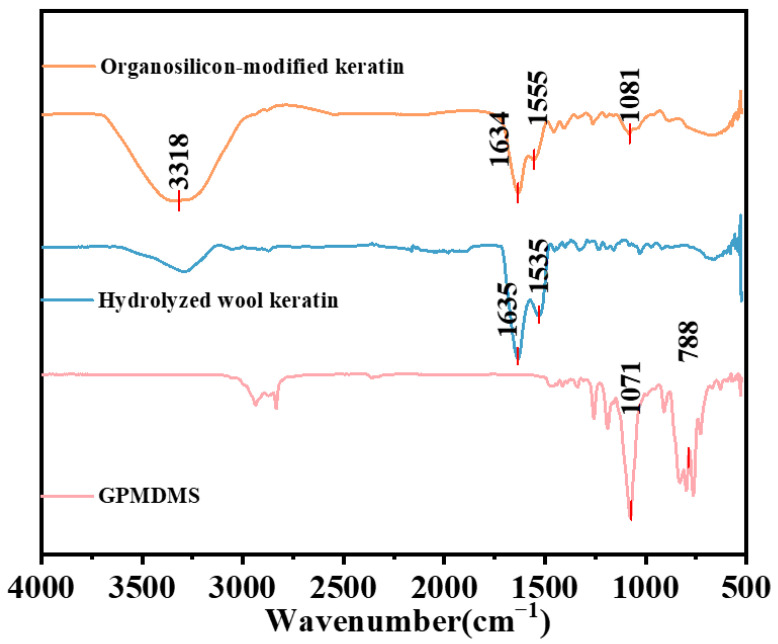
Infrared spectra of hydrolyzed wool keratin, GPMDMS and OSK.

As shown in Figure 2, FT-IR spectra confirmed the OSK remained the C–N signals (1550–1640 cm^−1^) associated with keratins but introduced distinct Si–O (1080.51 cm^−1^) stretching vibrations and broad O–H vibrations (3317.90 cm^−1^) [22]. These changes indicated that the silyl group has been successfully attached to the keratin.

**Figure 3:**
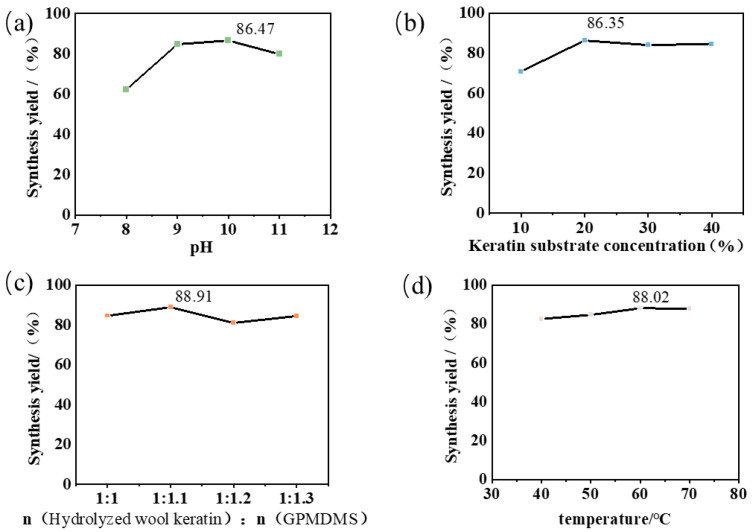
(**a**) pH and (**b**) keratin substrate centration and (**c**) reaction substrate molar ratio and (**d**) temperature on the efficiency of the OSK reaction.

Moreover, the effects of pH, substrate centration, molar ratio of hydrolyzed wool keratin to GPMDMS, and reaction temperature on the reaction yield were evaluated. It can be observed in Figure 3 that both the reaction pH and substrate concentration had significant effects on the product yield. As shown in Figure 3a, the system pH exhibited a distinct volcanic-shape curve relationship with the synthesis yield, achieving a maximum yield of 86.47% at pH 10. However, further increasing the pH lowered that yield, which could be ascribed to the acceleration of the side reactions like self-condensation of hydrolyzed silane molecules. As shown in Figure 3b, the concentration of keratin also affected the synthesis yield. When the keratin concentration was lower than 20%, increasing the concentration could significantly increase the yield of the reaction. However, when the keratin concentration was higher than 20%, there was no significant improvement in the conversion efficiency. This might be due to the fact that when the concentration is too high, steric hindrance and the shielding effect of internal amino groups limit the further improvement of the synthesis yield. Notably, reactant molar ratio and temperature variation exhibited slight effects on the synthesis yield. The optimal reaction conditions were ultimately determined to be pH = 10, a hydrolyzed wool keratin substrate concentration of 20%, molar ratio of 1:1.1, and a temperature of 60 °C. Under these conditions, the reaction yield reached 90.67%.

### 2.2. Effects of OSK on Hair Surface Properties

**Figure 4:**
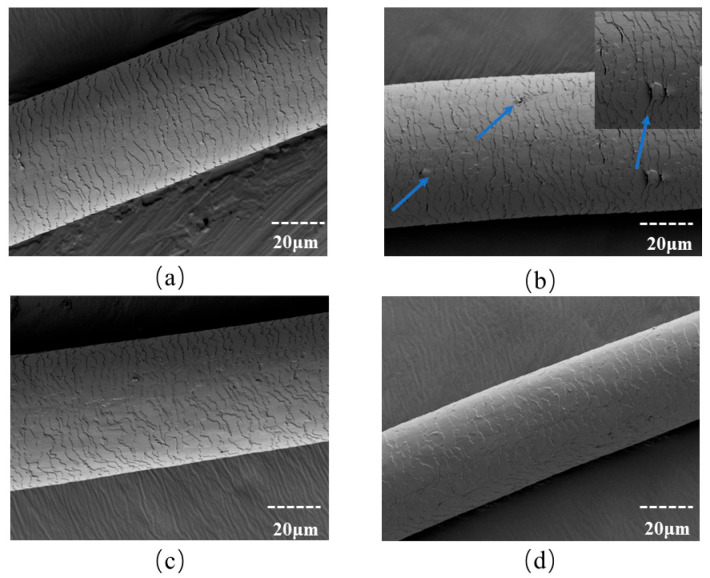
SEM images of (**a**) virgin hair, (**b**) heat-damaged hair, (**c**) OSK-repaired hair, and (**d**) OSK-prevented hair. The blue arrow indicates that the scales on the surface of the head are raised.

The structural integrity of the hair cuticle served as a key indicator of the overall health condition of the hair shaft, and one of the obvious characteristics of heat damage was the alteration of the morphology of the hair scales [23]. The efficacy of OSK in protecting hair cuticles against heat damage was evaluated using SEM. As shown in Figure 4a, the hair scales on the surface of virgin hair are orderly and closely arranged. The hair with thermal damage, shown in Figure 4b, exhibits obvious cracks and raised hair scales. In contrast, OSK-repaired heat-damaged hair demonstrates significantly improved surface morphology, characterized by reduced scale roughness and tightly adhered cuticles. More importantly, it was found that OSK-prevented hair, in which OSK treatment was applied before heating, could greatly protect hair morphology, which displayed a more ordered cuticle arrangement with a clearly visible continuous film-coating on the hair’s surface. These results indicated a thermal responsiveness of OSK, which probably undergoes some chemical changes upon heat activation.

**Figure 5:**
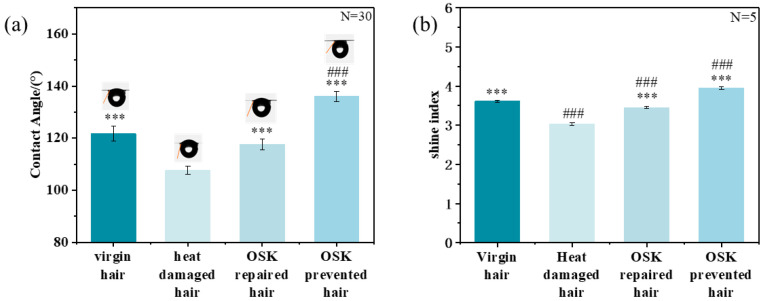
The contact angles (**a**) and gloss (**b**) of different hair samples. (* indicates comparison with heat-damaged hair. # Compared with virgin hair. Independent samples *t*-tests were used to determine whether there were significant differences. *p*: *** < 0.001, *p*: ### < 0.001). The picture in (**a**) represents the contact angle diagram.

The film-forming effect of OSK resulted in significantly improved hair hydrophobicity. As shown in Figure 5a, heat-damaged hair exhibited markedly lower hydrophobicity than virgin hair, with the contact angle decreased from 121° to 107°. In contrast, OSK-repaired hair exhibited a contact angle of 117°, approaching virgin-hair levels. More importantly, OSK-prevented hair demonstrated excellent heat-damage protection with a contact angle of 136°, exceeding virgin hair and meeting the high hydrophobicity standard of 120° to 150° [24,25]. Furthermore, the OSK-prevented hair exhibited superior gloss. In comparison, OSK-repaired hair showed a 13.48% increase in gloss, while OSK-prevented hair exhibited a 30.26% improvement compared to heat-damaged hair. Notably, OSK-prevented hair has an increased gloss level by 30.26% compared to virgin hair, indicating the protective effect of the OSK molecules on the surface of the hair, providing a better sensory effect.

**Figure 6:**
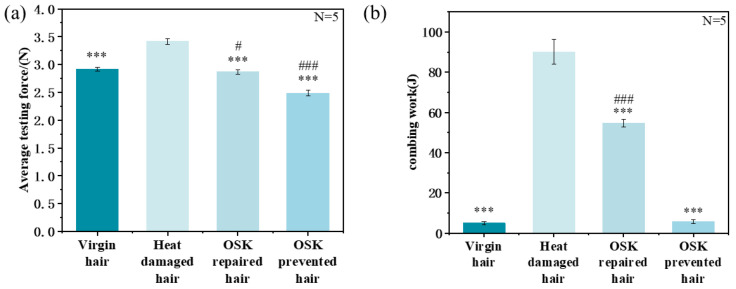
The (**a**) smoothness and (**b**) combing performance of several types of hair. (* indicates comparison with heat-damaged hair. # Compared with virgin hair. Independent samples *t*-tests were used to determine whether there were significant differences. *p*: *** < 0.001, *p*: # < 0.05, ### < 0.001).

Another advantage brought by highly hydrophobic surfaces is the improvement of smoothness and combing performance. OSK treatment significantly improved hair smoothness, with OSK-prevented hair demonstrating superior smoothness with 14.73% higher than virgin hair. As shown in Figure 6b, the combing work of OSK-repaired hair was significantly reduced by 35.38 J compared to heat-damaged hair. These results indicate that OSK could effectively protect the hydrophobic barrier of the hair and improve the sensory appearance of the hair.

### 2.3. Mechanism Investigation of Thermal Responsiveness

**Figure 7:**
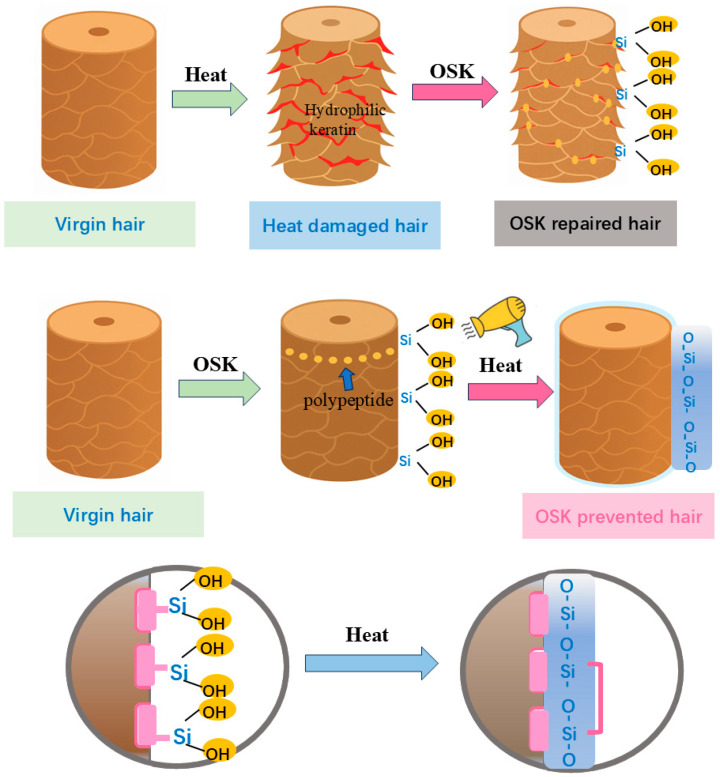
Mechanism of action of OSK hair care.

The above comparison of the repair performance for damaged hair and the protection performance for undamaged hair demonstrates that OSK could exhibit better performance effects after heating. It showed advantages over virgin hair in terms of the repair of the hair’s hydrophobic barrier, glossiness, and combability. This result was believed to be related to the thermoresponsiveness of OSK. The thermoresponsive behavior of OSK is believed to be rendered by the dehydration–condensation of its Si–OH groups under thermal activation, forming a continuous Si–O–Si network on the hair surface, as illustrated in Figure 7. During treatment with OSK, peptide fragments are adsorbed onto the hair surface, and thermally responsive groups are aligned in an ordered manner. Upon heating, these Si–OH groups underwent dehydration and condensation reactions, leading to the formation of a cross-linked siloxane network. This process resulted in a dense, continuous protective layer, as SEM observed. This coating enhanced the hair’s resistance to moisture penetration and thermal injury, while simultaneously contributing to the restoration of mechanical- and surface properties. This cross-linked structure exhibited enhanced thermal stability, which could be attributed to the high bond energy of Si–O bonds (460 kJ/mol), as reported in studies by Liu et al. [20] and Jia et al. [21]. The robust siloxane framework effectively improved the heat resistance of the modified keratin system.

**Figure 8:**
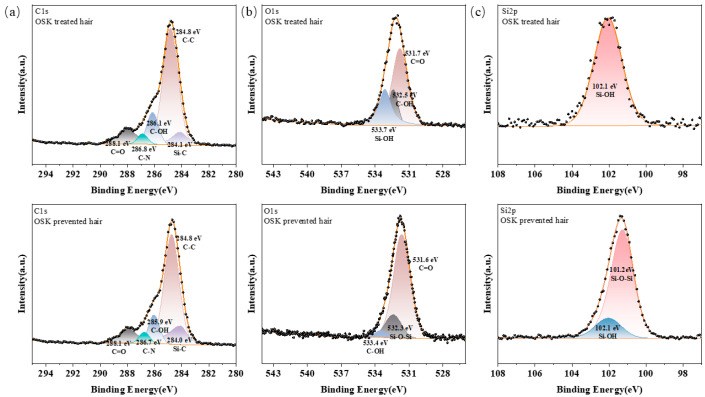
(**a**) C1s, (**b**) O1s, and (**c**) Si2p XPS spectra of OSK-treated hair and OSK-prevented hair.

To verify the above hypothesis, XPS was used to characterize the oxidation states of O, C, and Si elements in the OSK-treated hair and OSK-prevented hair. The high-resolution O1s XPS spectra could be resolved at 532.3 and 533.7 eV, which corresponded to the Si-O-Si and Si-OH groups, respectively [26]. The O1s spectrum in Figure 8b shows a new peak at 532.3 eV in OSK-prevented hair, corresponding to the formation of Si-O-Si bonds [19,20]. At the same time, changes in bond energy can also be observed in Si2p. As shown in Figure 8c, in OSK-treated hair, the bond energy of Si-OH was 102.1 eV, indicating that the amino-acid end of the OSK molecule successfully interacted with the hair. After thermal treatment, in OSK-prevented hair, a new peak of Si-O-Si could be seen with a bond energy of 101.2 eV [27]. Compared with the Si–OH peak, the binding energy was shifted to a lower value. This is because the hydrogen atom in -OH has very little electronegativity, which causes the oxygen atom that is connected to it to attract the electrons of the Si atom more strongly. Compared to the oxygen in Si-O-Si, the oxygen in Si-OH showed a stronger induction effect, while in Si-O-Si, the Si atom was connected by an oxygen bridge. Although oxygen was highly electronegative, the electron distribution in polysiloxane chains or cross-linked structures reaches a certain balance, resulting in the observed lower binding energy for Si–O–Si. This observation provides direct evidence that silicified keratin successfully forms a film on the surface of the hair, confirming that heating causes the dehydration condensation of Si–OH groups, resulting in a covalent cross-linked Si–O–Si network. This process is a chemical bonding change and is irreversible, which is consistent with the principle shown in Figure 7.

**Figure 9:**
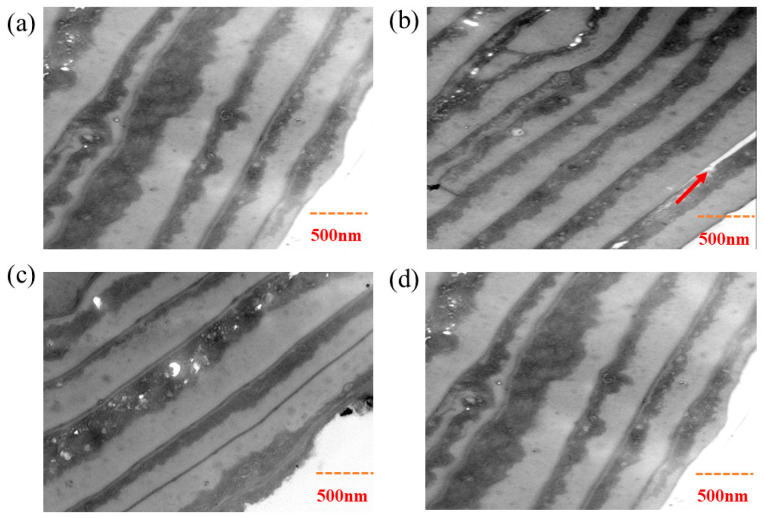
TEM images of ultrathin sections of keratinocytes from virgin hair (**a**), heat-damaged hair (**b**), OSK-repaired hair (**c**), OSK-prevented hair (**d**). The red arrow indicates that the bristles are raised.

The speculation of the above protection mechanism can also be confirmed by the TEM results. As shown in Figure 9b, the heat-damaged hair had its hair scales raised due to heat damage, and white vacancies appeared inside the lipid layer, indicating that CMC was also damaged during the heat-damage process and the surface hydrophobic layer was disrupted. The SEM results demonstrated that OSK exerts its protective effect by forming a membrane structure on the surface of the hair. In Figure 9d, the cross-section of the hair blocked by the OSK can also clearly show that the CMC of the hair is effectively protected, without any hair scales lifting up, thereby confirming the mechanism shown in Figure 7.

### 2.4. Effects of OSK on Hair Mechanical Properties

The lipids in the cuticle, cortex, and medulla layers of the hair shaft form a protective barrier that could resist damage to the hair from the environment and chemical factors, ensuring the hair flexibility. When the barrier is disrupted, the hair hydrophobicity changes, which will affect the mechanical properties of the hair strands [28].

**Figure 10:**
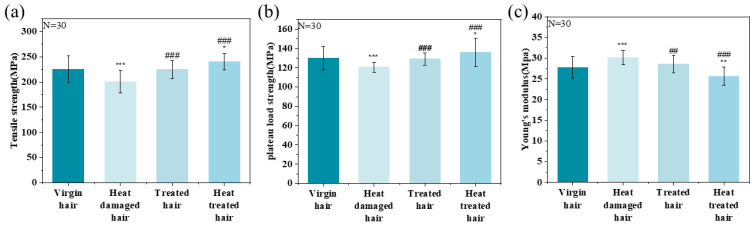
The effects of different treatment methods on hair samples, (**a**) tensile strength; (**b**) plateau load strength; (**c**) Young’s modulus (* indicates comparison with virgin hair. # Compared with heat-damaged hair. Independent samples *t*-tests were used to determine whether there were significant differences. *p*: * < 0.05; ** < 0.01, *** < 0.001, *p*: ## < 0.01, ### < 0.001).

As shown in Figure 10, the tensile strength, plateau load strength, and Young’s modulus were quantified. Compared to virgin hair, heat-damaged hair exhibited a 10.96% reduction (24.74 MPa) in tensile strength. Young’s modulus generally reflects the hardness or stiffness of the hair. Due to the external thermal environment, the hydrogen bonds were in an unnatural, tense state during cooling, causing the elasticity of the hair to decrease and for it to become stiff, and Young’s modulus also increases. Treatment with OSK significantly restored mechanical performance, increasing tensile strength by 23.72 MPa (11.80%) and yield force by 8.66 MPa (7.18%), while enhancing overall fiber stiffness and resilience. It was seen that OSK can effectively prevent the harm to hair caused by heat damage. The tensile strength increased by 14.85 MPa (6.58%), while plateau load strength rose by 6.05 MPa (4.65%), indicating enhanced resistance to permanent deformation. Concurrently, Young’s modulus decreased by 2.09 MPa (7.53%). This was because OSK is a heat-responsive keratin. The membrane structure formed on the surface of the hair in a high-temperature environment has a strong protective effect.

**Figure 11:**
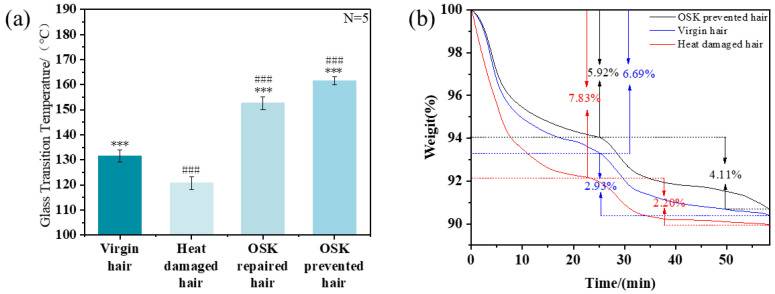
(**a**) Glass transition temperature and (**b**) the curve of the weight of hair strands over time of different hair samples (* indicates comparison with heat-damaged hair. # Compared with virgin hair. Independent samples *t*-tests were used to determine whether there were significant differences. *p*: *** < 0.001, *p*: ### < 0.001).

The glass transition temperature (Tg) of hair characterizes the critical point at which molecular mobility within the keratin matrix becomes significantly restricted. As shown in Figure 11a, OSK-treated hair exhibited a marked increase in Tg compared to virgin hair (131.6 °C), demonstrating enhanced thermal resistance. This improvement can be attributed to the continuous protective-film formed by OSK on the surface of the hair. This indirectly indicates that the OSK network forms and functions as a protective layer under high temperatures, with its effective activation range covering the daily thermal conditions ranging from 80 °C to 200 °C. This hydrophobic film acts as an effective barrier, reducing water loss from the hair when it is heated and enhancing the interaction forces between keratin molecules.

This improvement could be attributed to the continuous film formed by OSK on the hair surface, which restricted polymer chain mobility and raised the transition temperature. The protective layer thereby helped maintain structural integrity and barrier stability even under high-temperature exposure. The curve of the weight change in hair strands over time also proves the protective effect of this film structure. As shown in Figure 11b, the first mass decrease segment represents the step of removing free water. After the hair undergoes thermal damage, the hydrophobic barrier on the surface is disrupted and becomes hydrophilic, with the content of free water being 7.83%, which is 1.14% higher than that of virgin hair. After using OSK to protect the hair, due to the penetration of amino acids providing more and stronger water-binding sites, a large amount of bound water is locked in, with a content of 4.11%, which is significantly higher than that of damaged hair and virgin hair. And because it forms a protective film on the hair surface, it reduces the random adsorption of external water and reduces the loss of internal water, thereby enhancing its ability to fix water molecules. This indicates the excellent protective ability of the OSK molecules.

## 3. Materials and Methods

### 3.1. Reagents and Materials

Hydrolyzed wool keratin (98%) was procured from Shouhe Biotechnology Co., Ltd. (Xi’an, China) and 3-[(2,3)-Epoxypropyl] propyl methyldimethoxysilane (GPMDMS) was procured from Jiexika Chemical Co., Ltd. (Hangzhou, China). Sodium hydroxide, hydrochloric acid, and sodium dodecyl sulfate were procured from Sinopharm Chemical Reagent Co., Ltd. (Shanghai, China). The Amino Acid (AA) Content Assay Kit was procured from Boxbio. (Beijing, China). Virgin black Chinese hair (27 cm × 6 g) was purchased from Shanghai Canyu Commercial Co., Ltd. (Shanghai, China).

### 3.2. The Synthesis of OSK

Typically, a GPMDMS solution with 20wt% was hydrolyzed at 30 °C, with 1wt% citric acid added to the solution to keep the pH of 3.0. The mixture was stirred at a speed of 300 rpm in a water bath for 30 min. Then, a 20% hydrolyzed wool keratin solution was added drop by drop to the GPMDMS solution at 50 °C and the pH of the solution was adjusted to 10.0 using 1 wt% sodium hydroxide solution. After that, the mixture was centrifuged at 10,000 rpm for 10 min. The supernatant was concentrated via rotary evaporation to obtain final product. The yield of the product is determined by the conversion rate of the free amino groups. The change in free amino groups during the reaction was determined using the AA Content Assay Kit [29].

### 3.3. Hair Sample Treatment

Virgin black Chinese hair bundles were washed with a 10% (*w*/*v*) SDS solution to remove surface impurities before using [30].

Heat-damaged hair was obtained by straightening virgin hairs using a flat iron at 200 °C. Each heat treatment cycle simulated a typical straightening session, assuming that such behavior occurs 5 days out of 7 in a week. This is more in line with user habits, including three flat hairpin operations from the hair roots to the tips. Each operation, lasting for a total of 84 heating cycles, was conducted to simulate the usage over a year (52 weeks). This scheme draws on the common accelerated aging approach used in laboratory research, aiming to simulate the cumulative damage trend that may result from long-term and frequent use of flat iron. This enables the performance improvement brought by OSK to be stably observed and quantified, which is crucial for demonstrating its core mechanism of action. After heat treatment, the hair was not washed to retain all unstable keratins within the hair shaft.

OSK-repaired hair was obtained through a 30 min immersion of heat-damaged hair in 1% (*w*/*v*) OSK at 40 °C. The hair was blow-dried on a cool setting, rinsed with water for one minute, and dried again.

OSK-prevented hair was obtained by treating virgin hair with 1% (*w*/*v*) OSK at 40 °C for 30 min. The hair was blow-dried on a cool setting, rinsed with water for one minute, and dried again. Finally, the hair was heat-treated with a straightening iron to obtain the OSK-prevented hair.

### 3.4. Characterization of Hair

#### 3.4.1. FT-IR Spectroscopy

FT-IR of raw materials and OSK were recorded using a Nicolet iS50 spectrophotometer (Thermo Fisher Scientific, Waltham, MA, USA) in the range of 500–4000 cm^−1^. The structural characterization of several hair samples was analyzed using FT-IR.

#### 3.4.2. Differential Scanning Calorimetry (DSC)

The internal keratin structure changes in hair were investigated using DSC (NETZSCH Scientific, Selb, Germany). Approximately 5–10 mg of finely cut hair samples were placed into an aluminum DSC crucible (high-pressure 27 µL gold-plated, close). The crucible lid was perforated to ensure pressure equilibrium between the interior and exterior environments. In order to obtain the glass transition (Tg) of dry hair, hair fiber snippets (5–10 mg) were added to aluminum DSC pans and sealed with a perforated lid to allow water to evaporate during the measurement. Initially, the samples were heated at a rate of 10 °C/min to 200 °C, after which two further heating/cooling cycles were performed up to 150–200 °C [31].

#### 3.4.3. Thermogravimetric Analysis (TGA)

The internal moisture content of the hair was determined using a thermogravimetric analyzer (TGA), following the protocol established by Barba et al. [9]. Measurements were carried out under a controlled temperature program consisting of three stages. Initially, the samples were heated from 25 °C to 65 °C at a rate of 10 °C/min and held isothermally for 15 min. Subsequently, the temperature was increased to 185 °C at 10 °C/min and maintained for 20 min. Finally, the samples were heated from 185 °C to 205 °C at a heating rate of 10 °C/min, while the sample mass was monitored continuously throughout the process.

#### 3.4.4. Scanning Electron Microscopy (SEM)

The surface morphology of hair samples was characterized using SEM (Phenom ProX, Eindhoven, The Netherlands). The hair strands were affixed to aluminum pins with conductive carbon tape and were subsequently sputter-coated with gold to ensure sufficient conductivity. Observations were carried out at an acceleration voltage of 5 kV, and images were acquired at suitable magnifications to reveal surface features [32]. The image shown represents the representative morphology selected after observing at least 20 individual hair fibers in each group.

#### 3.4.5. Hair Hydrophobicity

To quantitatively evaluate the hydrophobicity of hair, contact angle tests between water droplets and hair fibers were conducted using an optical contact angle meter (OCA 40, Beijing Oriental Delphi Instrument Co., Ltd., Beijing, China) [33]. For each group of hair samples, 20 individual fibers were randomly selected, and a 2 μL distilled water droplet was suspended on each fiber using a micro-syringe. The average degree served as the quantitative indicator.

#### 3.4.6. Transmission Electron Microscopy (TEM)

TEM micrographs were acquired using a Zeiss Libra 120 microscope (UNICAMP Campinas—Sao Paulo, Brazil) operating at 120 kV. To examine cortical damage in hair cross-sections, samples subjected to various treatments were sectioned and stained for TEM analysis. Hair strands were fixed in 0.1 mol/L sodium oxalate buffer (pH 7.0) containing 2 wt% OsO_4_ for 4 h under dark conditions, followed by graded dehydration in ethanol solutions ranging from 50% to 100% (*v*/*v*). The dehydrated samples were embedded in Spurr resin, allowed to polymerize for 5 days, and then cured at 70 °C for 24 h. Ultrathin sections of 500 nm thickness were prepared using an ultramicrotome, and sequentially stained with 2 wt% uranyl acetate for 1 h and 1 wt% lead citrate for 15 min prior to TEM observation [34]. The figure shows the typical area images selected after observing 10 ultrathin sections prepared for each group of samples.

#### 3.4.7. X-Ray Photoelectron Spectroscopy (XPS)

XPS measurements were carried out using a SCIENTIFIC ESCALAB Xi+ instrument (Thermo Fisher Scientific, Waltham, MA, USA) equipped with a monochromatic and focused aluminum Kα X-ray beam. A single hair fiber was fixed on the sample holder with double-sided adhesive tape. All XPS measurements were performed using a focused X-ray source (diameter 400 µm; power 25 W) and 100 electron volts of photoelectrons at energy. Charge compensation of the sample was accomplished through a dual-beam charge compensation system. Binding energy values were corrected relative to the C1s peak at 284.6 eV [35].

### 3.5. Hair Performance Evaluation

#### 3.5.1. Tensile Property Tests

The mechanical properties of virgin, heat-damaged hair, OSK-repaired hair, and OSK-prevented hair were evaluated using an XS (08) XT-3 single-fiber strength tester (Shanghai Xusei Instrument Co., Ltd., Shanghai, China). Prior to mechanical testing, the diameter of each hair strand was measured using an LSM-501S laser (Mitutoyo, Tokyo, Japan) measurement system. Briefly, one end of the hair was positioned within the laser path, and the measurement was initiated via software control. The strand was then traversed slowly until the opposite end was reached, at which point the measurement was concluded. The system recorded both major- and minor axis diameters, and the average diameter was calculated from these values. For each group, 30 strands with diameters between 70 and 90 μm were selected for tensile testing. All experiments were performed under controlled environmental conditions (25 ± 2 °C, 50 ± 5% relative humidity) [36]. Tensile strength (*σ*), plateau load strength (${\bar{\sigma }}_{t}$), and Young’s modulus (*E*) were calculated using the following equations [37]:

(1) σ=F_b/S

(2) $${\bar{\sigma }}_{t}=(F\_h+F\_y)/2S,$$

(3) E=(F_H/S)/(∆L/L),

where *F*_*b* is the fracture strength, *S* is the cross-sectional area of the hair, Δ*L* is the displacement, *F*_*h* and *F*_*y* are the tensile forces at the end of the Hookean region and yield region, respectively, Δ*L* is the displacement, and *L* is the initial length.

#### 3.5.2. Hair Gloss

Hair gloss was measured on the Labmaster HAIR-GLOSS (Yanzhuang Biotechnology Co., Ltd., Shanghai, China) and calculated using the Reich–Robbins formula [38].

(4) L_(Reich−Robbins)=S/(D×θ_(1/2))

where *S* represents the total intensity of light in the specular reflection image; *D* represents the total scattered light intensity; and *θ*_1/2 represents the half-peak width of the light intensity distribution curve along the vertical axis in the specular reflection image. The larger the value of *L*, the more concentrated the light intensity, and the better the gloss of the hair.

#### 3.5.3. Hair Friction and Combability

Surface friction and combability of hair samples were quantitatively evaluated on a TSH-50 Multifunctional Hair Tester (Yanzhuang Biotechnology Co., Ltd., Shanghai, China), under controlled environmental conditions (25 ± 2 °C; 50 ± 5% relative humidity). Specifically, hair stands were fixed with adhesive tape above a silicone mold and allowed to hang freely, with the mold positioned 20 mm from the distal end. Before testing, the system was zero-calibrated with standard weights to offset the inherent weight of the hair and mold. The hair was then drawn root-to-tip at a constant speed of 200 mm/min. To reduce the effect of distal entanglement on friction readings, data were analyzed only within the 20–180 mm segment of the hair shaft. All measurements were automatically recorded and processed by integrated software to obtain frictional force results.

Combing force measurements were conducted using a fine-toothed comb fixture on vertically mounted hair samples and allowed to hang freely. After system warm-up, strands were evenly distributed in the comb, positioned 20 mm from the root section. The comb was drawn from root to tip at 200 mm/min. Data from the 20–180 mm segment along the hair shaft were analyzed to exclude distal entanglement effects. All measurements were automatically recorded and processed by the integrated software to obtain combing force results.

## 4. Conclusions

This study successfully synthesized a thermoresponsive OSK molecule by reacting hydrolyzed keratin with GPMDMS. XPS analysis confirmed that OSK undergoes thermal dehydration of hydroxyl groups to form a robust protective membrane on the hair surface. SEM and TEM verified this film prevents cuticle lifting and shedding under thermal stress. Notably, OSK treatment enhanced the hair’s hydrophobic barrier, achieving a 136° contact angle and increased glossiness by 30.26%. This indicates that the OSK not only have the key performance in the restoring of virgin hair but also provides excellent hydrophobic protection and sensory enhancement. The combing test results showed that after OSK treatment, the combing properties of heat-damaged hair could be improved by 35.38%. OSK also ensured the smoothness of hair during the heating process. Mechanical tests showed a 6.58% increase in tensile strength and a 7.53% reduction in Young’s modulus, indicating superior toughness and resistance to deformation.

## Data Availability

The original contributions presented in this study are included in the article. Further inquiries can be directed to the corresponding authors.
